# Neurosciences at Universiti Sains Malaysia Represent Malaysia to Support Global Precision Brain Health

**DOI:** 10.21315/mjms2019.26.3.1

**Published:** 2019-06-28

**Authors:** Jafri Malin Abdullah

**Affiliations:** Malaysian Journal of Medical Sciences, Universiti Sains Malaysia Health Campus, Kubang Kerian, Kelantan, Malaysia

**Keywords:** global, precision, brain, health, Malaysia, neurosciences, neurosurgery, psychology

## Abstract

The combined effort of the neuroscience and psychology cluster at the Universiti Sains Malaysia (USM)—fundamental, applied and clinical—has moved the institution to the number two position in the country, behind Universiti Malaya. The strategy to join the Global Brain Consortium (GBC) and put Malaysia on the map to address the GBC mission, vision, focus areas and outcomes began recently, in May 2019.

## Introduction

Malaysia was honoured to be invited to attend the World Health Organization (WHO)-supported Global Brain Consortium (GBC) workshop with the motto ‘Towards Global Precision Brain Health’ at the Montreal Neurological Institute’s de Grandpré Communications Centre in Montreal, Canada from 9–10 May 2019. The topics discussed ranged from the future expectations of the WHO in terms of global brain health, international and multilateral research-funding agencies, data-sharing infrastructures, and data governance for Electroencephalography (EEG) to studying dynamic brain starts with EEG, clinical research opportunities for EEG, clinical care translation using EEG, and the role of funders in advancing global brain collaborations.

## USM Moves to the International Level

The GBC is a diverse network of brain researchers, clinicians and institutions committed to achieving improved and more equitable health outcomes worldwide. It is a Canada-based initiative that builds collaborative and dynamic relationships among global-oriented health researchers, domestically and internationally.

The GBC will address the challenges facing multinational collaborative efforts in support of Global Precision Brain Health. It aims to strengthen linkages between like-minded neuroscientists across borders and disciplines to build a fluid and connected global research community that can advance equitable solutions to priority health challenges worldwide, with an orientation towards brain health and research challenges in low- and middle-income countries (LMICs) and guided by the overarching goal of reducing disparities in health outcomes (1).

Malaysia has prepared itself by establishing training programmes via Universiti Sains Malaysia’s graduate-level courses that consider the United Nations’ Sustainable Development Goals, especially in education. Global brain health requires trained medical and non-medical personnel combining both the arts and the sciences (2–6).

**Figure 1:**
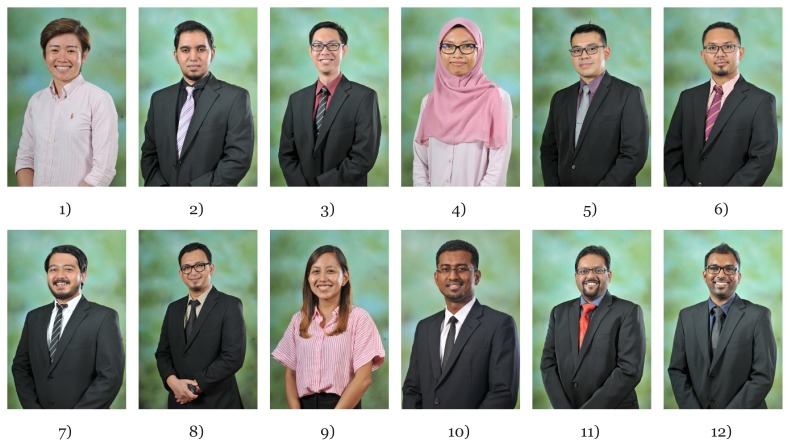

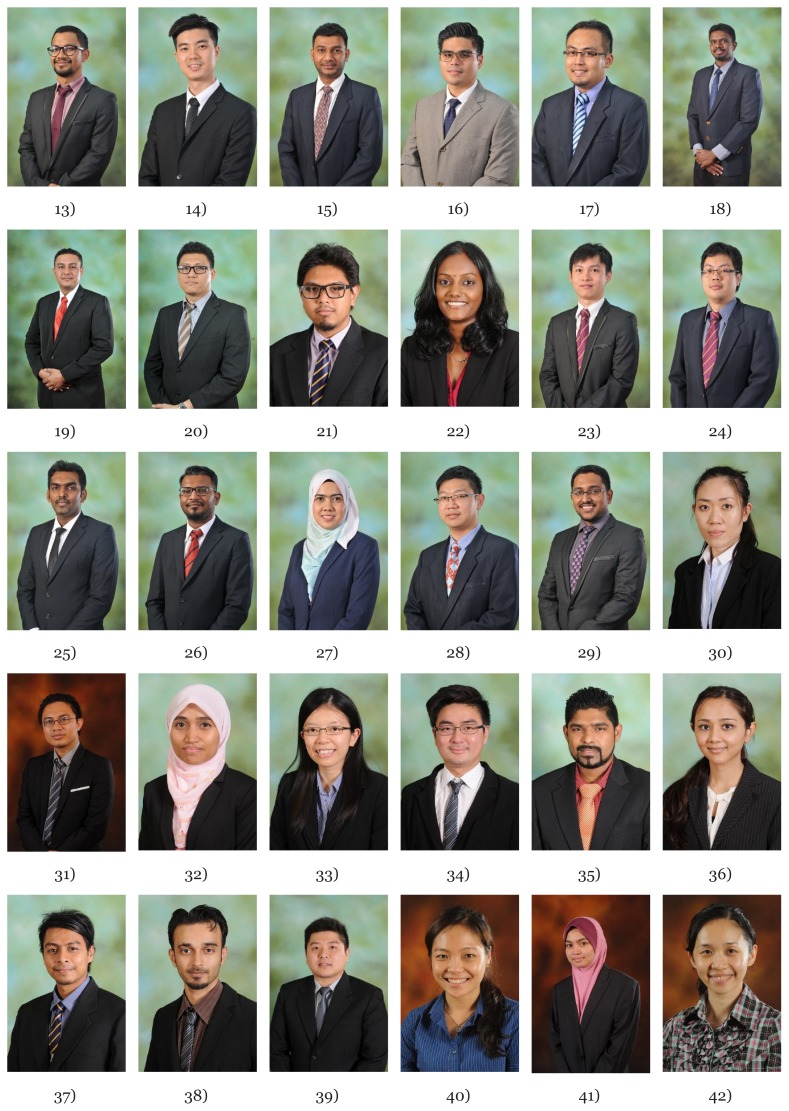

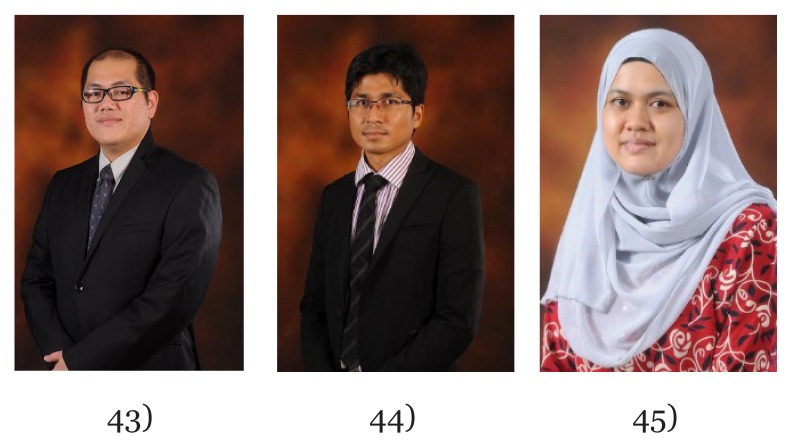
Master of Surgery (Neurosurgery) residents till May 2019 Year 1 1) Dr Debbie Kong Ching Ching 2) Dr Harvinth Nagalingam Muniandy 3) Dr Looi Mun Choon 4) Dr Mas Syazaneeza Shab 5) Dr Mohd Farhan Mohd Faiz Wilson Yeo 6) Dr Mohd Ghadafi Wahab 7) Dr Mohd Iryan Che Othman 8) Dr Muhamad Ridzuan Alias 9) Dr Nadiah Ahmad Fuad 10) Dr Nishan Rao Subramaniam 11) Dr Rohan Jeevaraj 12) Dr V Jeyaseelan G Vasanthakumaran 13) Dr Vicnesh Thillynathan 14) Dr Zaharul Azran Zahari Year 2 15) Dr Is Shahrom 16) Dr Julian Tan Li Kwang 17) Dr Kuha Raj a/l Arumugam 18) Dr Mohd Hezry Abu Hassan 19) Dr Mohd Khairun Mohd Mispan 20) Dr Thavanesan a/l Puvanesavaran Year 3 21) Dr Ahmad Zulfadli Mohamed Radzi 22) Dr Alarmelu Nithya Ramanathan 23) Dr Ang Song Yee 24) Dr Jesse Ze Ngai 25) Dr Kumarappan a/l Chokalingam 26) Dr Moventhiran a/l Ramakrishnan 27) Dr Nurshaheda Mohd Salleh 28) Dr Sam Joe Ee 29) Dr Saravanan a/l Sridharan 30) Dr Lim Mei Sin 31) Dr Mohd Najmi Abd Halim 32) Dr Sarah ‘Atiqah Mohd Zamri 33) Dr Tan Shze Ee 34) Dr Teo Eu Gene Year 4 35) Dr Arulkanesh Devatathan 36) Dr Diana Noma Fitzrol 37) Dr Muhammad Aizzat Othman 38) Dr Razmeender Singh Kelly 39) Dr Tan Zi Han 40) Dr Lee Chun Lin 41) Dr Shukriyah Sulong 42) Dr Lee Shwu Yi 43) Dr Lau Bik Liang 44) Dr Asrarul Fikri Abu Hassan 45) Dr Asmaa Mohamad Afifi

**Figure 2:**
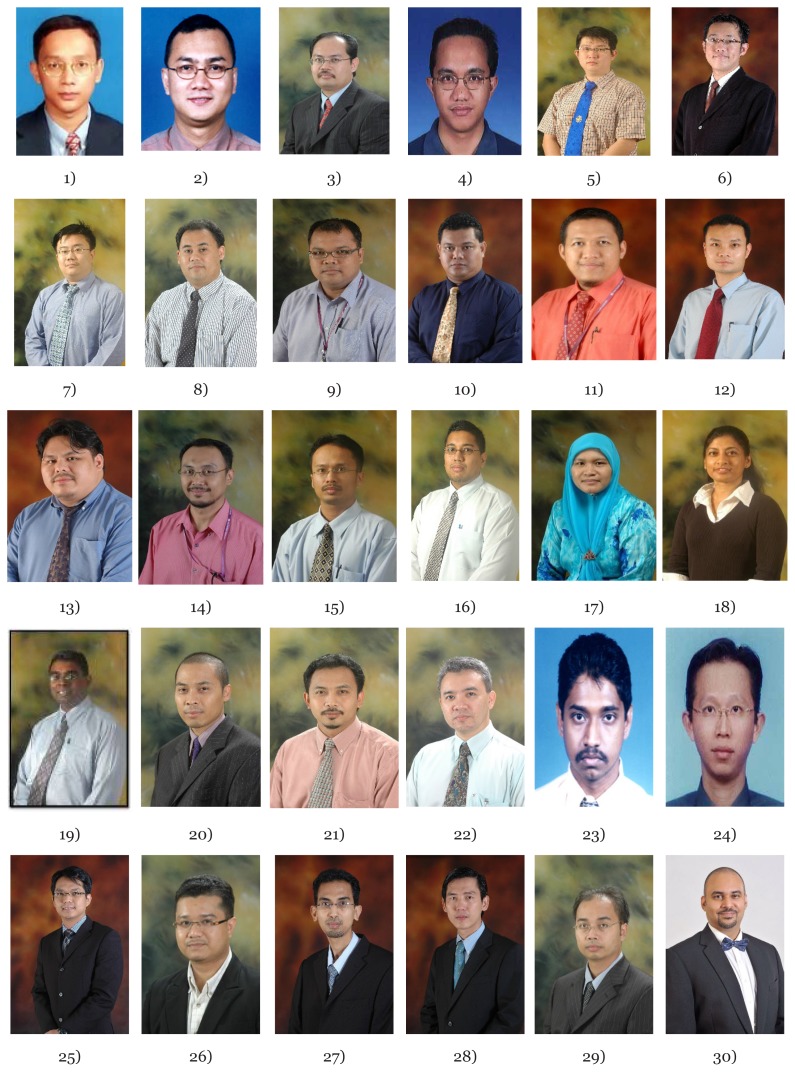

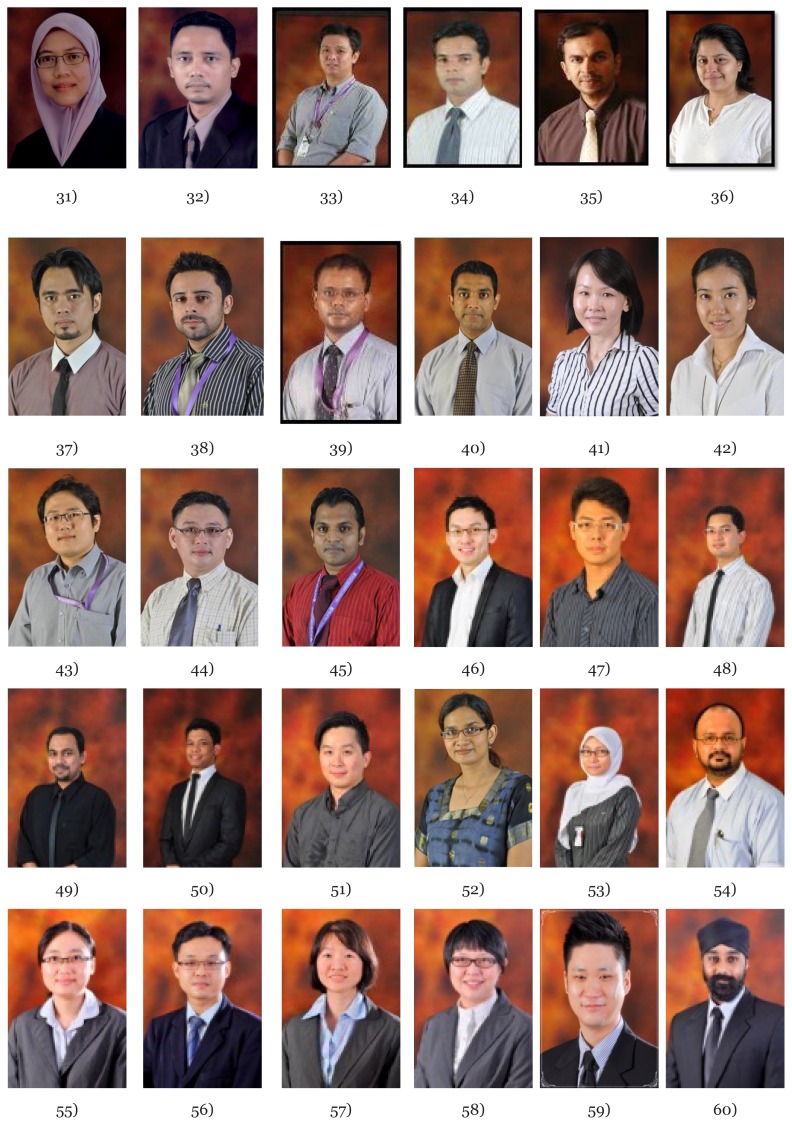

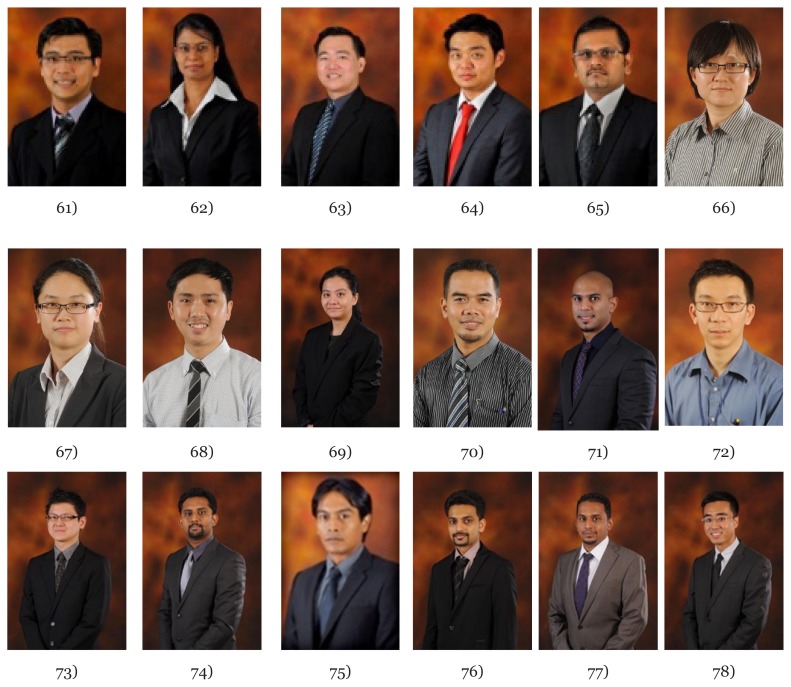
Neurosurgeons who have graduated from the postgraduate neurosurgical programme since 2001 Remember your neurosurgical academic lineage: My neurosurgical teacher was Professor Luc Calliauw from Hospital University Ghent, Belgium who was taught by Professor Henk Verbiest from University Hospital Utrecht, Holland who was taught by Professor Clovis Vincent from Pitié-Salpêtrière Hospital, Paris, France who was taught by Professor Joseph Jules Francois Felix Babinski from the same hospital in France and the famous Professor Harvey Cushing of Harvard Medical School, USA 1) Professor Dr Zamzuri Idris 2) Associate Professor Dato’ Dr Abdul Rahman Izaini Ghani 3) Dr Badrisyah Idris 4) Dr Adam Mohd Zakaria 5) Dr Kan Choon Hong 6) Dr Lee Hock Keong 7) Dr Gee Teak Shang 8) Dr Mohd Saufi Awang 9) Dr Farizal Fadzil 10) Dr Rahmat Harun @Haron 11) Dr Hafiz Mohd Zain 12) Dr Chan Kin Hup 13) Dr Sofan Zenian 14) Dr Saiful Razman 15) Dr Ahmad Zamzuri Remeli 16) Dr Mohd Aidil Mohd Noor 17) Dr Siti Suriyati Buang 18) Dr Sharon Casilda Theophilus 19) Dr Premananda Raja a/l Murugesa 20) Dr Sani Sayuthi 21) Dr Saiful Azli 22) Dr Naseer Abdul Wahab 23) Dr Kantha a/l Rasalingam 24) Dr Toh Charng Jeng 25) Dr Tan Wei Ming 26) Dr Nujaimin Udin 27) Dr Mohd Azhari Omar 28) Dr Liew Boon Seng 29) Dr Adrean Husin 30) Dr Regunath a/l Kandasamy 31) Dr Risdhawati Hasan 32) Dr Asraf Sharifudin 33) Dr Lim Swee San 34) Dr Mohammad Azman Raffiq 35) Dr Gerard Arvind Martin 36) Dr Priya Sharda Jagdish Mitter 37) Dr Faizul Hizal Ghazali 38) Dr Puneet Nandrajog 39) Dr Kamalanathan a/l Palaniandy 40) Dr Thinesh Kumaran 41) Dr Cheah Pooi Pooi 42) Dr Siti Azleen Mohamad 43) Dr Ch’ng Chee How 44) Dr Sim Sze Kiat 45) Dr Ananda Arumugam 46) Dr Mah Jon Kooi 47) Dr Tan Yew Chin 48) Dr Mohd Raffiz Mohd Ali 49) Dr Jason Raj a/l Johnson Kovilpillai 50) Dr Fadzlishah Johanabas Rosli 51) Dr Adrian Ng Wei Chih 52) Dr Jacintha Vikeneswary 53) Dr Ailani Ab Ghani 54) Dr Senthil Kumar a/l Rajapathy 55) Dr Ng Wei Peng 56) Dr Ariz Chong Abdullah @ Chong Chee Yong 57) Dr Low Siaw Nee 58) Dr Low Yong Lee 59) Dr Lim Liang Hooi 60) Dr Manvinder Singh Mangat 61) Dr Lai Chuang Chee 62) Dr Kanmani Dewi 63) Dr Chan Chee Kong 64) Dr Mohd Syahiran bin Mohd Sidek 65) Dr Prabu Rau a/l Siram 66) Dr Low Peh Hueh 67) Dr You Xinli 68) Dr Goh Chin Hwee 69) Dr Neoh Yee Yik 70) Dr Muhammad Ihfaz Ismail 71) Dr Davendran Kanesen 72) Dr Kho Giat Seng 73) Dr Nelson Yap Kok Bing 74) Dr Rajendra Rao a/l Ramalu 75) Dr Rakesh Rethinasamy 76) Dr Ramissh Paramasivam 77) Dr Vinodh a/l Vayara Perumall 78) Dr Yee Sze Voon

**Figure 3:**
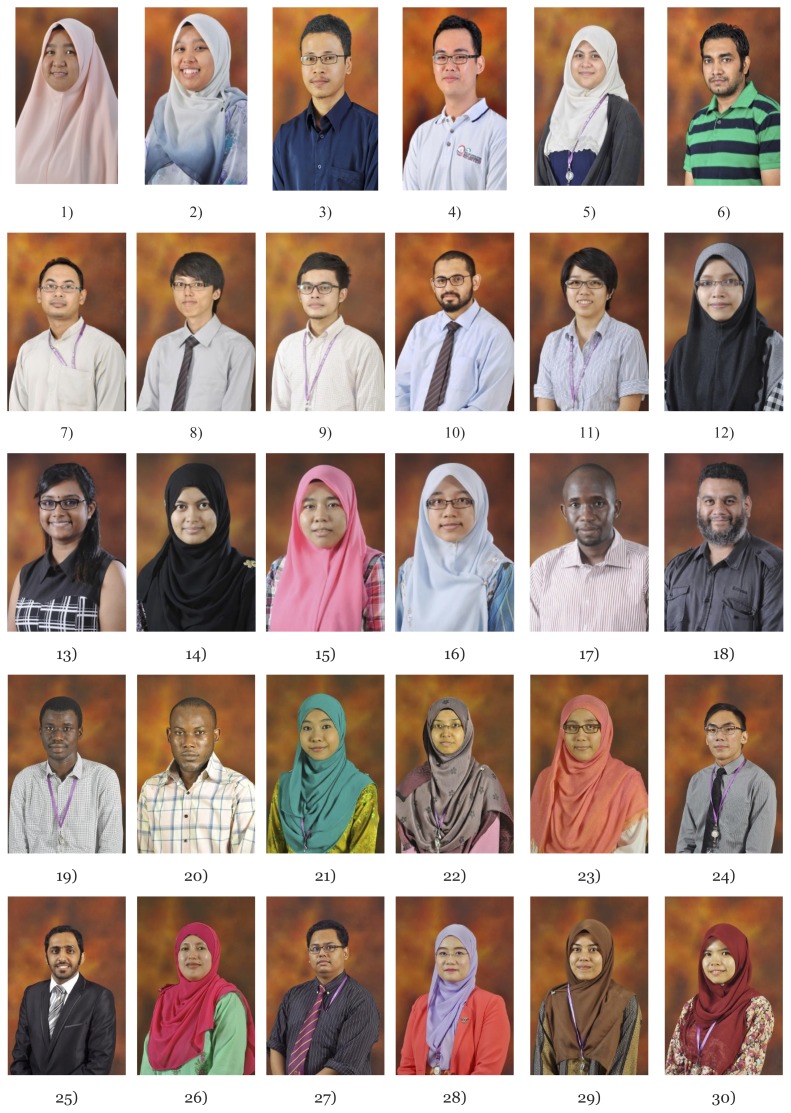

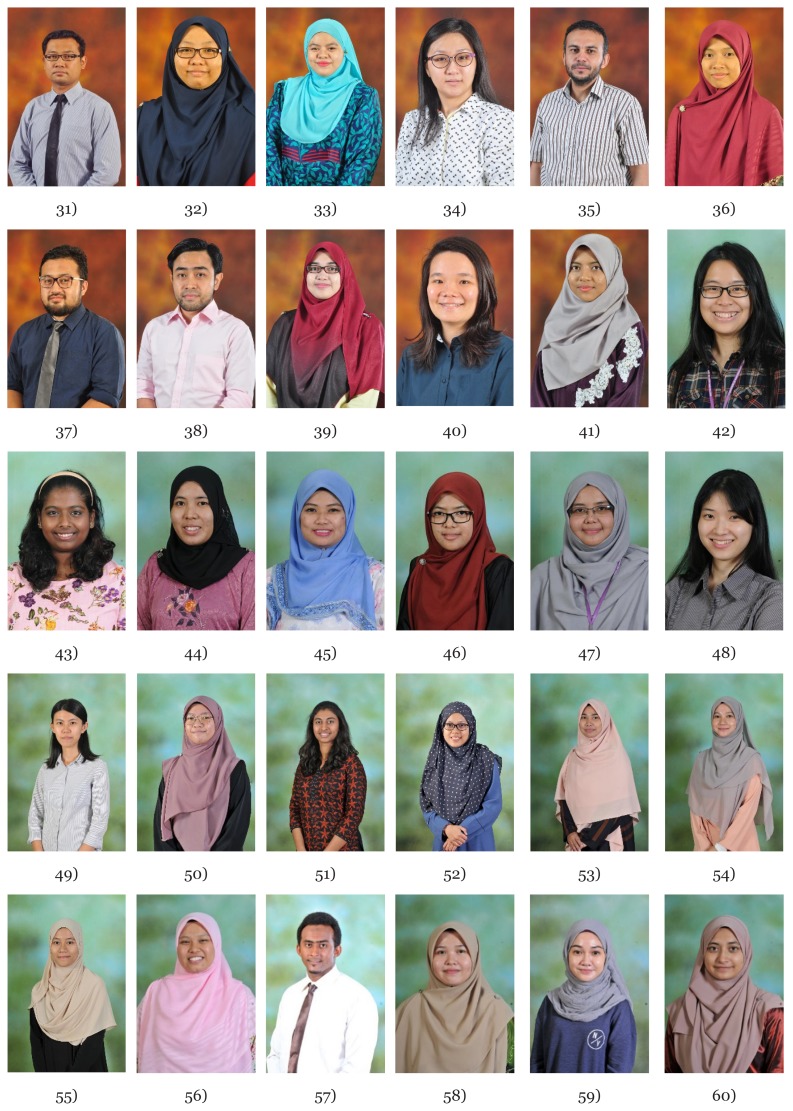

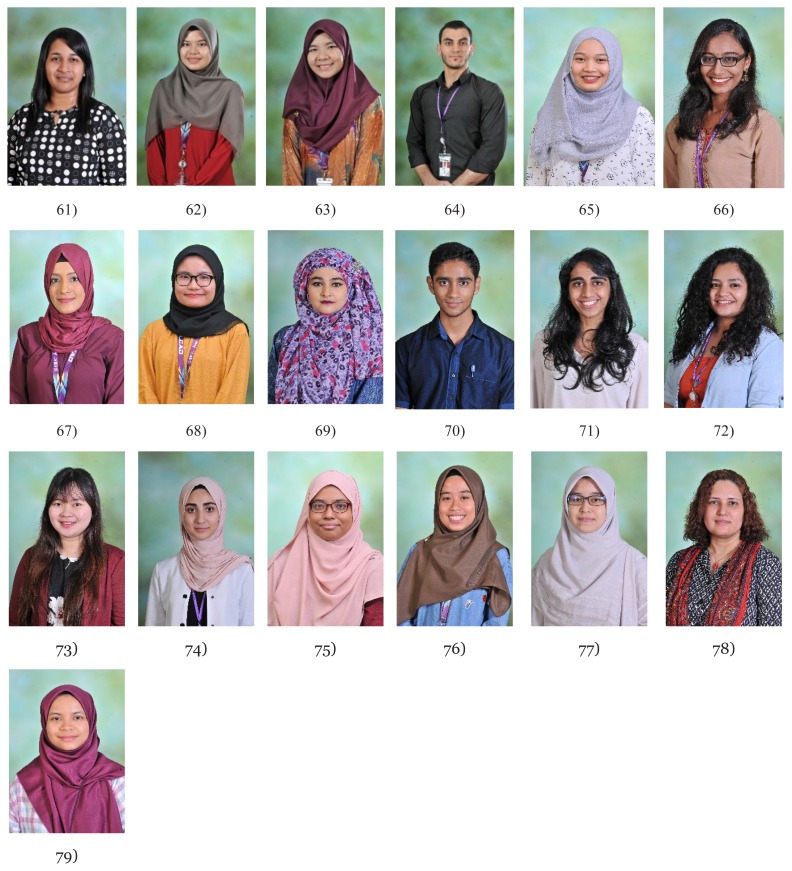
Integrated neuroscience programme students from 1st batch till 11th batch 1st batch INP students 1) Dr. Wan Mohd Daud Wan Omar 2) Nazirah Hanim Sharipudin 3) Rumaisa Abu Hassan 4) Teo Yong Chang 2nd batch INP students 5) Al-Zahrani Hussain Saad 6) Che Mohd Nasril Che Mohd Nasir 7) Koh Junhao 8) Mohd Ibrahim Abdullah 9) Muhammad Bilal 10) Nazeerah Abd Rahman 11) Wong Jia Hui 3rd batch INP students 12) Saidah Napisah Muhammad 13) Priatharsine Seerangan 14) Nurul Fatihah Othman 15) Nur Aimi Zawami Ahmad 16) Fatin Hilyani Mohamad 17) Ahmad Adamu Adamu 18) Usman Jaffer 4th batch INP students 19) Abu Bakar Tijjani Salihu 20) Auwal Bello Hassan 21) Norlyiana Samsuri 22) Rafidah Rosli 23) Siti Nur Ain Zakaria 5th batch INP students 24) Chuang Huei Gau 25) Faraj Almarri 26) Mazira Mohamad Ghazali 27) Muhammad Afiq Mahayidin 28) Nor Aqilah Mohd Yusuf Yeo 29) Nurfaizatul Aisyah Ab Aziz 30) Nurul Atikah M Nor Nazli 31) Shazlan Noor Suhaimi 6th batch INP students 32) Amanina Ahmad Safri 33) Amy Shafinas Azman 34) Chong Pei Nei 35) Khaled Sayed Rabia Elsayed 36) Mas Syazwanee Shab 37) Mohd Khairul Izamil Zolkefley 38) Mohd Waqiyuddin Abdullah 39) Nadia Izzati Nordin 40) Tai Yan Shan 41) Ummi Nasrah Talib 7th batch INP students 42) Chai Wen Jia 43) Ivanna Fernandez 44) Nur Adila Ramli 45) Nur Naznee Hirni Abd Aziz 46) Nurfarhana Abdul Ghani 47) Siti Atiyah Ali 48) Tan Celina 8th batch INP students 49) Cheeh Hui Lee 50) Dayang Yasmin Abang Abdul 51) Revathy Murali 52) Siti Hajar Mohd Zaki 53) Siti Nurulhusna Hashim 54) Tiara Ramli 55) Zakiyyah Munirah Mohd Zaki 9th batch INP students 56) Aishah Sakinah Zahid 57) Mohammed Abdalla Kannan Ahmed 58) Nor Azulaikha Abdullah 59) Nurul Nazihah Zaidil 60) Sara Fatini Abdul Naser 61) Sri Ratha Balakrishnan 10th batch INP students 62) Farah Madihah Mustafa Kamal 63) Nurul Amirah Mohammad Murad 64) Younis MS Firwana 11th batch INP students 65) Anis Raihan Dzeidee Schaff 66) Devi AP Ananth 67) Hadjer Khiati 68) Iffah Syafiqah Suhaili 69) Iman Imtiyaz Ahmed Juvale 70) Joshua Kuruvilla 71) Kiirtaara Aravindhan 72) Malvika Sharma 73) Nornadirah Abdul Rahman 74) Nour Mohammed Abdallah Qaddumi 75) Nur Nasihken Mawi 76) Nurdarina Ausi Zulkifli 77) Nurulain Syahirah Razali 78) Rabia Nazir 79) Wan Shahirah Wan Adnan

**Figure 4:**
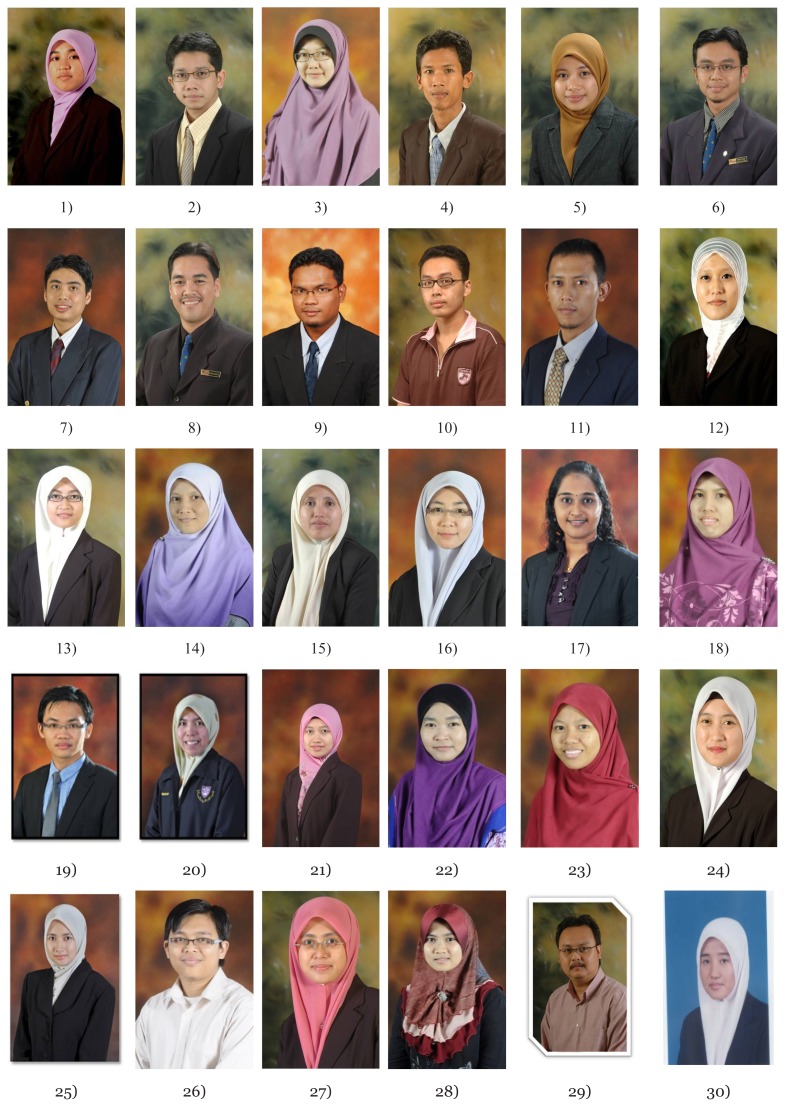

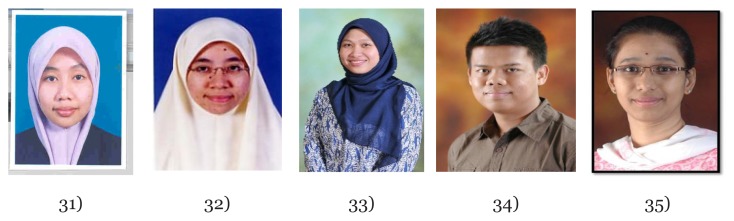
Master by Pure Research, Doctorate by Pure Research and Advanced Master of Medicine (Neurology) Neurosciences 1) Siti Norhajah Hashim 2) Mohd Azim Patar 3) Putri Nur Hidayah Al-Zikri Mohamad Akil 4) Dr. Zulkifli Mustafa 5) Raisah Abd Hadi 6) Mohd Nasir Mat Nor 7) Syed Fariq Fathullah Syed Yaacob 8) Mohd Harizal Senik @ Nawi 9) Mohd ‘Ulul ‘Ilmie Ahmad Nazri 10) Ahmad Tarmizi Che Has 11) Muhammad Hanif Che Lah 12) Norshazrin Shazira Shafee 13) Siti Zawani Mohd Ramli 14) Wan Noor Ainun Baharuddin 15) Mazira Mohamad Ghazali 16) Samhani Ismail 17) Nanthini a/p Jayabalan 18) Wan Salihah Wan Abdullah 19) Tee Jong Huat 20) Khalilah Haris 21) Khairol Naaim Mohd Nasir 22) Nur Syairah Abd Rani 23) Mashytah Abdul Karim 24) Nor Entan Supeno 25) Nurul Iman Wan Ismail 26) Asyraf Abd Rahman 27) Emmilia Husni Tan 28) Siti Zulaikha Nashwa Mohd Khair 29) Abdul Aziz Mohamed Yusoff 30) Farizan Ahmad 31) Norafiza Zainudin 32) Sarina Sulong 33) Siti Naziha Hasma Hassan 34) Zarif Sofian Neurology 35) Dr Shalini Bhaskar

The Master of Surgery (Neurosurgery) programme of the Universiti Sains Malaysia is training future neurosurgeons (Figure 1) and has thus far graduated 78 neurosurgeons (Figure 2) since this 2-plus-4-year postgraduate coursework was initiated in 2001, currently coordinated by Professor Zamzuri Idris, Professor Dato’ Dr Jafri Malin Abdullah, Associate Professor Abdul Rahman Izaini Ghani and Dr Regunath Kandasamy. The established Integrated Neuroscience Programme (INP), coordinated by Dr Muzaimi Mustapha, is on its eleventh batch of postgraduate students, producing both Masters and Doctors of Neurosciences since its establishment five-and-a-half years ago (Figure 3). The Master of Neurosciences by pure research, master coursework in neurology, and Doctorate in Neurosciences currently coordinated by Dr Farizan Ahmad has produced more than 35 graduates (Figure 4).

**Figure 5:**
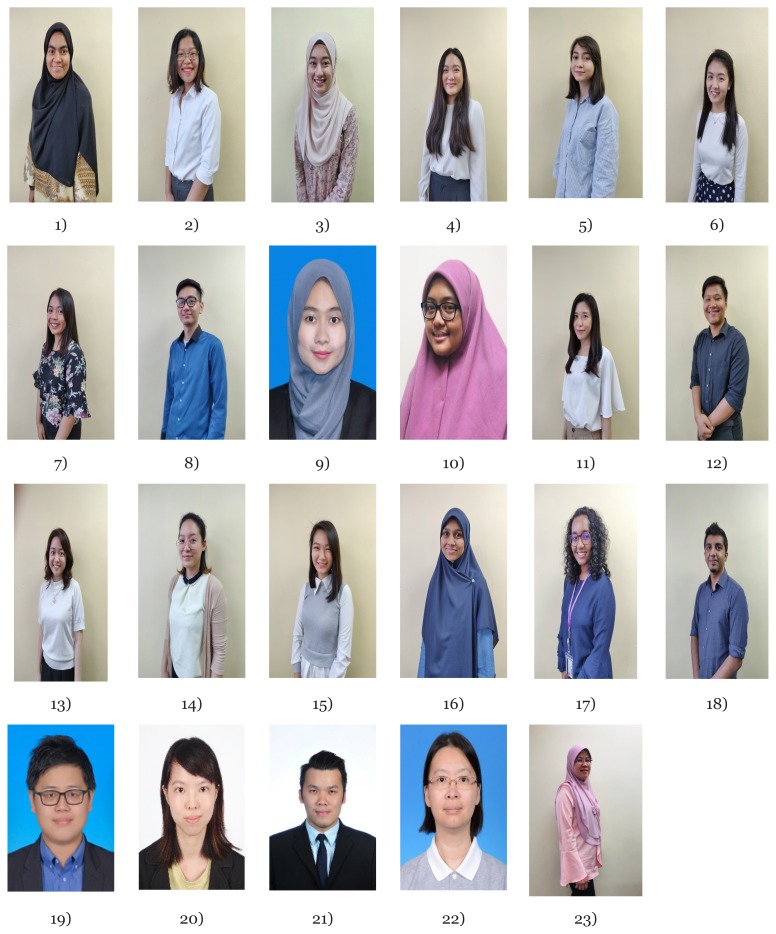
First batch Master of Clinical Psychology students USM-UPSI 1st batch Master Integrated Psychology Programme-USM 1) Ummi Norliyana Zainal 2) Angela Chen Aun Kei 3) Siti Amirah Hanna Jafri Malin 4) Audrey Poh Sze Huey 5) Sharifah Diana Syed Abdul Halim 6) Loh Ken Joey 7) Mariah Hanna 8) Mohamad Farahan Huszaimi M Pajar 9) Norsuhana Emilinadiah Husin 10) Nurulhidayah Mohd Saat 11) Seeh Ti Whan 1st batch Master Integrated Psychology Programme-UPSI 12) Muhammad Lutfi Md Nizam 13) Neo Jia Yeh 14) Norasyikin Jane Mustafa Kamal 15) Chao Thung Yin (Joyce) 16) Asmirah Jafarulla Khan 17) Suwarna Sukumaran 18) Abd Raouf Abdul Razak 1st batch Doctorate Integrated Psychology Programme-USM 19) Tay Kok Wai (Clinical Neuropsychology) 20) Michelle Choong Poh Kin (Clinical Psychology) 21) Liang Yaw Wen (Clinical Psychology) 1st batch Doctorate Integrated Psychology Programme-UPSI 22) See Geok Lan (Clinical Neuropsychology) 23) Atiqah Chew Abdullah (Clinical Psychology)

**Figure 6:**
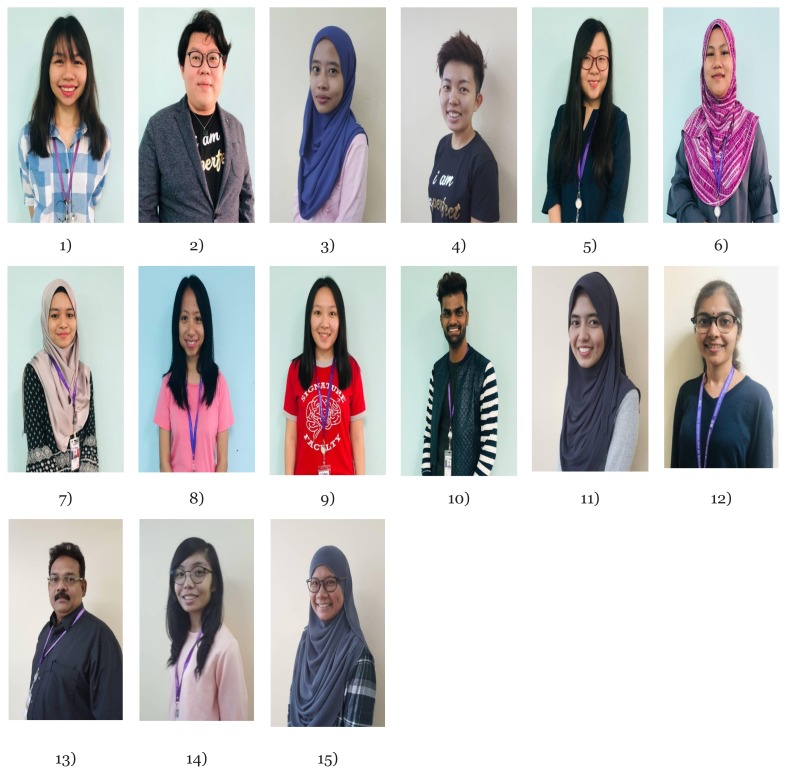
First and second batch of Master of Cognitive Neurosciences USM offered at Postgraduate Institute@Kuala Lumpur 1st batch 1) Joice 2) Wang Shi-Jie 3) Nurul Bayti Sumardi 4) June Lew Wan Ling 5) Chin Jia Wei 6) Norrul Aikma Mohamed 7) Khairiah Razali 8) Kartina Ismail 9) Lim Hui Sean 10) Yogendren Murugia 11) Nadiah Mohd Sukarno 2nd batch 12) Thilageswary a/p Doraisamy 13) Ramesh Kumar a/l Ramachandren 14) Audrey Antoine a/p Max Antoine 15) Eizan Azira Mat Sharif

**Figure 7:**
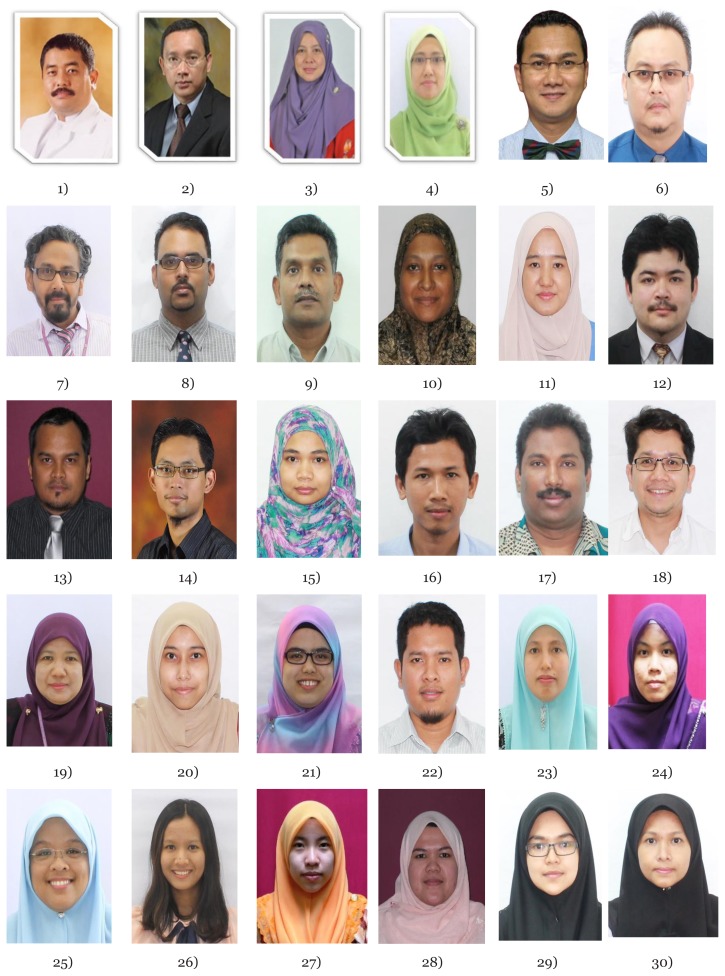

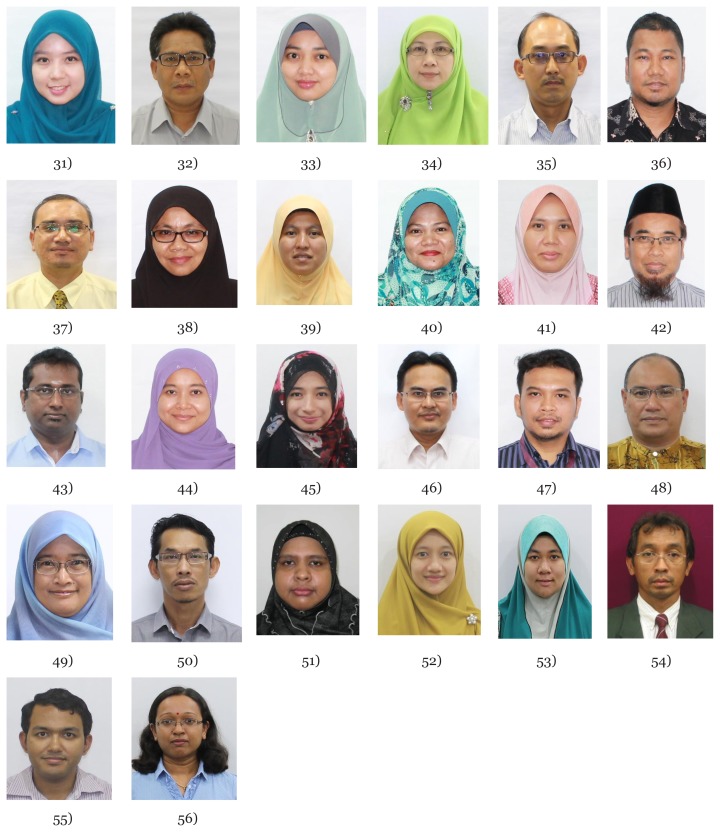
Members of Brain and Behaviour Cluster, [previously known as P3Neuro (Pusat Perkhidmatan dan Penyelidikan Neurosains)], School of Medical Sciences, Universiti Sains Malaysia 1) Professor Dato’ Dr Jafri Malin Abdullah-Chairman 2) Professor Dr Zamzuri Idris–Deputy Chairman 3) Associate Professor Madya Dr Asrenee Ab. Razak-Deputy Chairman 4) Associate Professor Dr Azizah Othman-Deputy Chairman 5) Associate Professor Dato’ Dr Ab Rahman Izaini Ghani @ Ab. Ghani 6) Associate Professor Dr Abdul Aziz Mohamed Yusoff 7) Associate Professor Dr Muzaimi Mustapha 8) Dr Regunath a/l Kandasamy 9) Dr Mohammed Faruque Reza 10) Dr Tahamina Begum 11) Dr Farizan Ahmad 12) Dr Muhammad Hafiz Hanafi 13) Dr Mohd Nasir Che Mohd Yusoff 14) Dr Mohamed Faiz Mohamed Mustafar 15) Dr Aini Ismafairus Abd Hamid 16) Dr Mohd Zulkifli Mustafa 17) Dr Sangu Muthuraju 18) Dr Mohd Nor Azim Ab Patar 19) Dr Sabarisah Hashim 20) Ms Nurul Aini M Nashir 21) Ms Nur Amalina Hashim 22) Mr Hazim Omar 23) Mdm Mazira Mohamad Ghazali 24) Mdm Alwani Liyana Ahmad 25) Mdm Wan Nor Azlen Wan Mohamad 26) Ms Kee Sui Mei 27) Mdm Nuraza Othman 28) Ms Nur Azza Husna Ahmad Satar 29) Mdm Nur Faten Hamzah 30) Mdm Sharifah Aida Shekh Ibrahim (Research Officer)) 31) Mdm Athirah Raihanah Abdul Wahab (Science Officer) 32) Mr Mohd Eilham Yusof 33) Dr Sanihah Abdul Halim 34) Associate Professor Dr Salmi Ab Razak 35) Associate Professor Dr Zahiruddin Othman 36) Dr Mohd Azhar Mohd Yasin 37) Dr Maruzairi Husain 38) Dr Norzila Zakaria 39) Dr Sharifah Zubaidiah Syed Jaapar 40) Dr Nor Asyikin Fadzil 41) Dr Raishan Shafini Bakar 42) Associate Professor Dr Mohd Shafie Abdullah 43) Dr Chandran a/l Nadarajan 44) Dr Nik Fariza Husna Nik Hassan 45) Cik Aimi Syahidah Zulkipli 46) Dr W Mohd Nazaruddin W Hassan 47) Dr Mohamad Hasyizan Hassan 48) Professor Dr Wan Hazabbah Wan Hitam 49) Professor Dr Rosni Abdullah @ Mustafa 50) Associate Professor Dr Putra Sumari 51) Dr Nurul Hashimah Ahamed Hassain Malim 52) Dr Nur Intan Raihana Ruhaiyem 53) Dr Nur Syibrah Muhamad Naim 54) Professor Dr Mohd Zaid Abdullah 55) Associate Professor Dr Haidi Ibrahim 56) Dr Anusha a/p Achuthan

**Figure 8:**
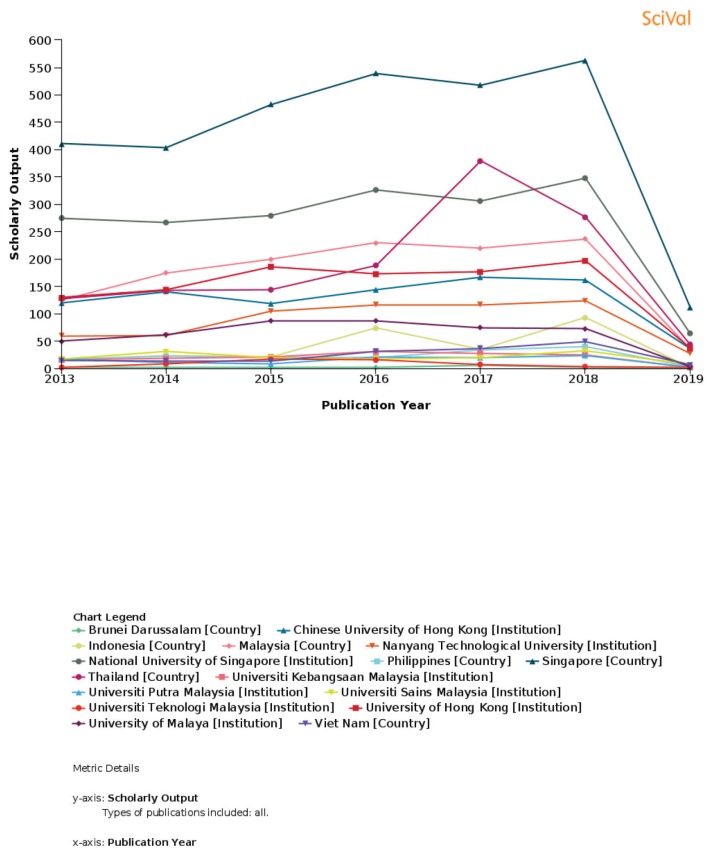
USM ranking in neuroscience according to SciVal, Elsevier First ranking: Universiti Malaya Second ranking: Universiti Sains Malaysia Third ranking: Universiti Kebangsaan Malaysia Fourth ranking: Universiti Putra Malaysia Fifth ranking: Universiti Teknologi Malaysia

**Figure 9:**
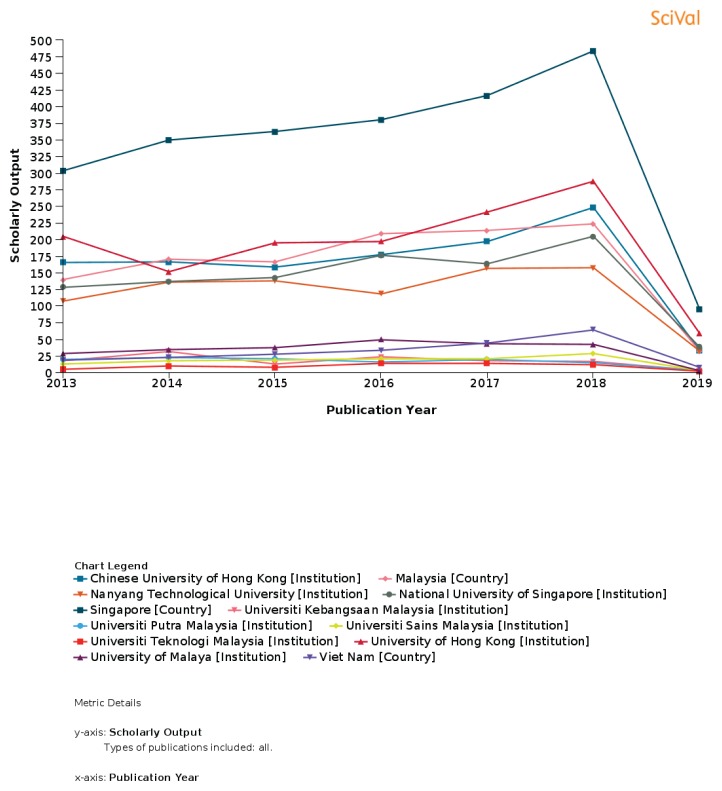
USM ranking in psychology according to SciVal, Elsevier First ranking: Universiti Malaya Second ranking: Universiti Sains Malaysia Third ranking: Universiti Kebangsaan Malaysia Fourth ranking: Universiti Putra Malaysia Fifth ranking: Universiti Teknologi Malaysia

**Figure 10:**
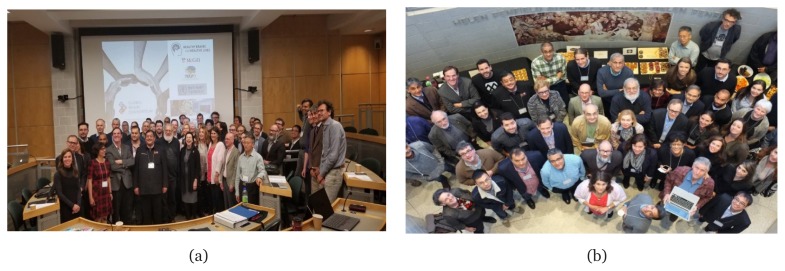
Pictures show Professor Alan Evan (a) front row, stood third from left, (b) at the back, stood second from left] and Professor Pedro Valdes Sosa (a) front row, stood fifth from left, (b) forth row, stood fifth from right], and the founders involved in the consortium

The newly established Integrated Clinical Psychology USM-UPSI courses coordinated by Associate Professor Dr Azizah Othman and Dr Mohamed Faiz Mohamed Mustafar and the Master of Cognitive Neuroscience courses coordinated by Professor Dato’ Dr Jafri Malin Abdullah and Dr Aini Ismafairus Abd Hamid received their first batches of students in September 2018 and the second batch of Cognitive Neuroscience students in February 2019 (Figures 5 and 6). The newly established Brain Behaviour Cluster (Figure 7) has jumpstarted its activities, focusing on translational, transdisciplinary excellence in clinical neurology, neurosurgery, and psychology, with at least 56 members in various fields. Thus, we hope the ranking of clinical neurosciences and psychology will improve over the next three years in Malaysia, as per Figures 8 and 9. The Global Brain Consortium that Malaysia was invited to join will further focus on collaboration with other under developed and near developed countries to improve their neurological and mental healthcare using neurotechnology like the EEG (Figure 10).

## What We Strive For

Mental and neurological disorders account for an increasing proportion of the global burden of disease. Additionally, the accessibility and affordability of appropriate treatment remains low, especially in countries classified as LMIC by the World Bank, where approximately 85% of the world’s population resides. To improve mental health services, LMICs must increase their workforces, particularly the number of trained professionals who can provide mental health services. Although primary health care professionals can provide the bulk of care, mental health professionals—namely psychiatrists, nurses and psychosocial health experts—are needed to manage patients who are referred for specialised care and to deliver training, support and supervision to non-specialists. Without these mental health professionals, LMICs will be unable to meet their populations’ mental health treatment requirements. Advanced technicians would also be involved in the development of EEG policies, procedures, provision of EEG-related supervision, and training of less-experienced EEG technicians and staff (2–6).

## Conclusion

The neuroscience group, in collaboration with psychiatry and psychology, hopes to further the global WHO agenda and envisions its implementation in the next three years. We hope for support from the Academy of Sciences Malaysia, universities, the Malaysian Ministry of Health and the public to make this endeavour a success.

The teacher who is indeed wise does not bid you to enter the house of his wisdom but rather leads you to the threshold of your mind—Khalil Gibran.
